# SCovid v2.0: a comprehensive resource to decipher the molecular characteristics across tissues in COVID-19 and other human coronaviruses

**DOI:** 10.1128/spectrum.01933-24

**Published:** 2024-12-23

**Authors:** Zijun Zhu, Xinyu Chen, Guoyou He, Rui Yu, Chao Wang, Changlu Qi, Liang Cheng

**Affiliations:** 1 College of Bioinformatics Science and Technology, Harbin Medical University, Harbin, Heilongjiang, China; 2 NHC Key Laboratory of Molecular Probe and Targeted Diagnosis and Therapy, Harbin Medical University, Harbin, Heilongjiang, China; Shandong First Medical University, Jinan, Shandong, China

**Keywords:** COVID-19, single-cell RNA-seq, bulk RNA-seq, other human coronaviruses, molecular characteristics

## Abstract

SCovid v2.0 (http://bio-annotation.cn/scovid or http://bio-computing.hrbmu.edu.cn/scovid/) is an updated database designed to assist researchers in uncovering the molecular characteristics of coronavirus disease 2019 (COVID-19) across various tissues through transcriptome sequencing. Compared with its predecessor, SCovid v2.0 is enhanced with comprehensive data, practical functionalities, and a reconstructed pipeline. The current release includes (i) 3,544,360 cells from 45 single-cell RNA-seq (scRNA-seq) data sets encompassing 789 samples from 15 tissues; (ii) the addition of 62 COVID-19 bulk RNA-seq data comprising 1,688 samples from 12 tissues; (iii) incorporation of seven bulk RNA-seq data sets related to other human coronaviruses, such as HCoV-229E, HCoV-OC43, and MERS-CoV for a thorough comparative analysis of pan-coronavirus mechanisms in COVID-19; and (iv) systematic comparisons between the data sets conducted using standardized procedures. Furthermore, we have developed an advanced search engine and upgraded web interface to browse, search, visualize, and download detailed information. Overall, SCovid v2.0 is a valuable resource for exploring molecular characteristics of COVID-19 across different tissues.

**IMPORTANCE:**

This manuscript provides a comprehensive analysis of the molecular characteristics of COVID-19 through cross-tissue transcriptome analysis, contributing to the understanding of COVID-19 by clinicians and scientists. Considering the cyclical nature of coronavirus outbreaks, this updated database adds transcriptome data on other human coronaviruses, contributing to potential and existing mechanisms of other human coronaviruses.

## INTRODUCTION

Since the beginning of the outbreak, coronavirus disease 2019 (COVID-19), caused by severe acute respiratory syndrome coronavirus 2 (SARS-CoV-2), represents a serious global public health crisis (1). Even though effective vaccines have successfully reduced both viral transmission and disease burden (2), essential questions remain about the COVID-19 pathophysiology. Among them is the urgent need to elucidate the molecular mechanism of COVID-19 to develop efficient treatments.

With in-depth case studies, accumulating evidence indicates that COVID-19 could affect multi-tissue involvement in the body. Although SARS-CoV-2 primarily infects the respiratory tract and produces pathological manifestations in the lung, patients with COVID-19 present with a wide range of disease indications, such as the gastrointestinal (3), cardiovascular (4, 5), and neurological systems (6). Concurrently, insight into cellular tropism and host responses has been gained, but certain key aspects underlying the infection regulation remain undetermined (7). In particular, it is unknown whether fundamental regulators could be the host response to SARS-CoV-2 infection across distinct tissue types, which would be a tremendous benefit for disease treatment with multi-systemic manifestations.

In 2022, the initial version of the SCovid database (SCovid v1.0) was released following a comprehensive review of available resources to reveal the molecular characteristics of COVID-19 (8). As the knowledge about COVID-19 has advanced, the emphasis has switched to the molecular characterization of COVID-19 under transcriptomics, which is not just single-cell RNA-seq (scRNA-seq) but is equally crucial for understanding COVID-19, the host, and their interactions. Comparing transcript levels between healthy controls and COVID-19 individuals, as the most explanatory power, allows for identifying differentially expressed genes (DEGs), which may be causes, consequences, or mere correlates of the disease under scrutiny. Moreover, there is some convergence in the molecular mechanisms of other human coronaviruses and COVID-19. For instance, a recent study discovered a significant boost in cross-reactive neutralizing antibodies in some patients exposed to MERS-CoV and SARS-CoV-2 antigens (9). These findings could guide the development of a pan-coronavirus vaccine by targeting cross-reactive epitopes between distinct strains of human coronaviruses.

We thoroughly revised the SCovid database to overcome the aforementioned problems, utilizing a unified pipeline of transcriptional alterations caused by SARS-CoV-2 infection in various cell types and tissues. First, we reconstructed the single-cell analysis pipeline in version v2.0, which includes cell- and sub-type cluster analyses, differential expression analysis, cell communication, and cell differentiation. Compared with SCovid v1.0, we manually collected 45 scRNA-seq data sets across 15 tissues and 62 bulk RNA-seq data sets across 12 tissues, significantly boosting data quantity and quality. Importantly, we added seven RNA-seq data sets on other human coronaviruses to compare with COVID-19. After that, we developed a bulk RNA-seq pipeline, thus further investigating the molecular mechanisms of COVID-19, and provided interactive charts for viewing. Finally, to optimize the search system and enhance the user experience, we introduced advanced filtering options and upgraded the system interface for aesthetic and minimalist design.

## MATERIALS AND METHODS

### Data collection

#### COVID-19 RNA-seq

We manually searched COVID-19-related RNA-seq data sets in SRA (10), PubMed, and GEO (11) according to the keywords: (“COVID-19” OR “SARS-CoV-2”) AND (“single cell” OR “single-cell”) AND (“bulk RNA-seq”) AND (“transcriptomics” OR “transcriptome” OR “scRNA-seq” OR “scRNA seq” OR “bulk RNA-seq”). We obtained the manual extraction of COVID-19-related literature and host information from publications. Before January 2024, we collected 45 scRNA-seq data sets from 15 tissue types and 62 bulk RNA-seq data sets from 12 tissue types.

#### Other human coronavirus RNA-seq

We manually searched other human coronavirus-related RNA-seq data sets in electronic databases, including SRA, PubMed, and GEO, based on the keywords: (“HCoV-229E”) AND (“HCoV-OC43”) AND (“HCoV-NL63”) AND (“HCoV-HKU1”) AND (“SARS-CoV”) AND (“MERS-CoV”). Before January 2024, we obtained seven bulk RNA-seq data sets from three virus species.

### Construction of scRNA-seq pipeline

#### Data processing

The raw data were each processed with Cell Ranger (version 6.1.2). Considering the technical noise of the assay, low-quality cells and low-expressed genes were removed by the following selection criteria: (i) cells with <200 genes, as well as genes expressed in <3 cells; (ii) cells that contained >20% of mitochondrial genes. The “NormalizeData” function was used to normalize the expression level for each cell with default parameters. The “ScaleData” function was employed to scale the expression of each gene. The scRNA-seq pipelines integrated the COVID-19 scRNA-seq data in a project, which enabled various cell- and sub-types in normal and COVID-19 tissues.

#### Dimensional reduction and cluster analysis

The “FindVariableFeatures” function was used to calculate variable genes with default parameters. Then, PCA analysis was performed using variable feature genes, and the principal components (PCs) identified by the function “ElbowPlot” were used to cluster the data set. Next, each cluster annotation was confirmed by our previous knowledge of known cell type-specific gene markers, obtained from DEGs of each cluster by the “FindMarkers” and “FindAllMarkers” functions. The “FindNeighbors” and “FindClusters” functions for t-distributed stochastic neighbor embedding (tSNE) clustering were implemented with customized dimension numbers and clustering resolutions.

#### Cell type sub-population analysis

Cells previously annotated were subset and re-clustered using the methods described above. Cell sub-populations were identified by highly variable genes and known maker genes from the original articles and the collected publications.

#### Differential expression analysis

We performed the analysis of scRNA-seq expression. First, for each cell- and sub-type, MAST (v1.16.0) was used to calculate DEGs between the cells from samples with COVID-19 and the cells from controls. Then, up-/downregulated genes with top 5% |Log_2_FC| and *P* < 0.05 were regarded as significant DEGs visualized by volcano plot.

#### Gene ontology (GO) and Kyoto Encyclopedia of Genes and Genomes (KEGG) analysis

The GO pathways and KEGG analysis of each cell- and sub-type were performed using the R package “clusterProfiler” (12). The threshold of significance (*P*-value) corrected by the Benjamini–Hochberg procedure was <0.05.

#### Developmental trajectory inference

For differentiation trajectory analysis, Monocle (version 2.14.0) algorithm with the signature genes from the “differentialGeneTest” function was used (13). The differentiation trajectory of selected cells was inferred with the default parameters. The pseudo-time-related genes were calculated by the “differentialGeneTest” function, and dot plots for the selected genes were generated using the Monocle function “plot_genes_in_pseudotime.

#### Cell communication

Putative cell–cell interactions between COVID-19 and healthy were quantified using CellphoneDB v2 with default settings (14). Seurat-normalized counts and sub-cluster annotation for each cell were input into CellphoneDB v2 to determine the potential ligand–receptor pairs.

### Construction of bulk RNA-seq pipeline

#### Data processing

The bulk RNA-seq data were processed using standard pipelines, including quantification of gene expression and quality control (QC). For bulk RNA-Seq data sets, the raw counts submitted in the study were converted into transcripts per million (TPM) values for comparison between data sets, and it was also retained and served as input to DESeq2 later. The gene names of expression profiles were unified as the HUGO gene symbol.

#### Pseudo-cell ratio analysis

To quantify the extent of infiltration at the COVID-19, other human coronaviruses, and control site, the R package “CIBERSORT” deconvolution algorithm was applied to estimate the proportions of immune cells. The LM22 signature gene file was selected for the background gene set.

#### Differential expression analysis

After the data standardization and normalization using the normalize between arrays function in the R package “limma” (15) and “DESeq2” (16), we performed differential expression analysis of COVID-19 and other human coronaviruses with controls. The screening criteria were determined as follows: (i) *P* adj <0.05 and (ii) |Log_2_ FC| > 2.

#### GO and KEGG analysis

To explore the potential biological function, the R package “clusterProfiler” was employed to perform the GO function annotation and KEGG pathways analysis of these up-/downregulated significant DEGs. The threshold of significance corrected by the Benjamini–Hochberg procedure was <0.05.

### Comparison of gene expression across data sets

SCovid v2.0 converts raw count data from scRNA-seq and bulk RNA-seq into TPM data, enabling comparability between data sets. Here, we use the R package “BioMart” to calculate the effective length of each gene and convert the counting data into TPM data. With TPM, we can more easily compare the proportion of readings mapped to genes in each sample, providing a reference for comparison of expression across data sets.

### Database implementation

SCovid v2.0 was constructed by Vue (v3.2.45), Spring Boot (v2.5.1), and MySQL (v5.7.24) for the Frontend–Backend Separation Project. As the frontend framework, Vue was responsible for page visualization and interaction with backend data. SpringBoot was used for backend business logic and data processing. MySQL was used as a data storage and query interface. HTML5, CSS, Axios, jQuery, TypeScript, and Element-Plus were used for web rendering and interaction. Echarts were used to draw interactive charts. Vxe-table is used to display tables on websites. R scripts were implemented for online bioinformatics analysis.

## RESULTS

### Data records

**Fig 1:**
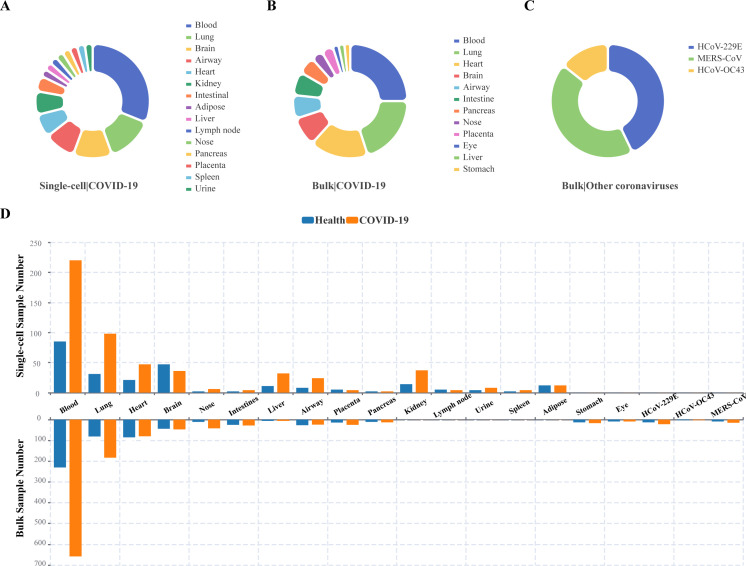
Statistics of projects and samples among organisms. Data set statistics for different tissues in (**A**) COVID-19|single-cell RNA-seq; (**B**) COVID-19|bulk RNA-seq; and (**C**) other human coronaviruses|bulk RNA-seq. (**D**) The experimental and control group statistics for single-cell RNA-seq and bulk RNA-seq data.

SCovid v2.0 contains 3,544,360 high-quality cells from 789 samples in 45 data set protocols, including 15 tissue types consisting of blood, brain, heart, urine, kidney, nose, placenta, pancreas, spleen, lymph nodes, liver, adipose, lung, intestine, and airway (Table 1). Additionally, the database gathers 62 data sets from 1,268 samples across 12 tissues for COVID-19, including blood, lung, pancreas, heart, stomach, eye, liver, brain, intestine, airway, nose, and placenta (Table 1). We also incorporate seven datasets from 65 samples that contain cells from other human coronaviruses (Table 1). The “Statistics” module contains further information (Fig. 1), and the “Download” page of SCovid v2.0 lists all the data.

**TABLE 1:** Comparison between v1.0 and v2.0 of the SCovid database

Data type	No. tissue types (v1.0, v2.0)	No. data sets (v1.0, v2.0)	No. samples (v1.0, v2.0)	No. cells (v1.0, v2.0)
Single-cell|COVID-19	(10, 15)	(21, 45)	(233, 789)	(1,042,227, 3,544,360)
Bulk|COVID-19	(-, 12)	(-, 62)	(-, 1,688)	(-, -)
Bulk|Other human coronaviruses	(-, -)	(-, 7)	(-, 65)	(-, -)

To explore the landscape of COVID-19 gene expression profiles in SCovid v2.0, we constructed the following five modules: (i) browsing each data set and multiscale analysis results; (ii) using multiple visualization techniques to show the genes expressed in a data set; (iii) comparing the gene expression across data set types; (iv) performing a functional enrichment analysis of significantly DEGs, and (v) viewing the patterns of gene expression and interactions in scRNA-seq data sets.

### User interface

#### Overview

**Fig 2:**
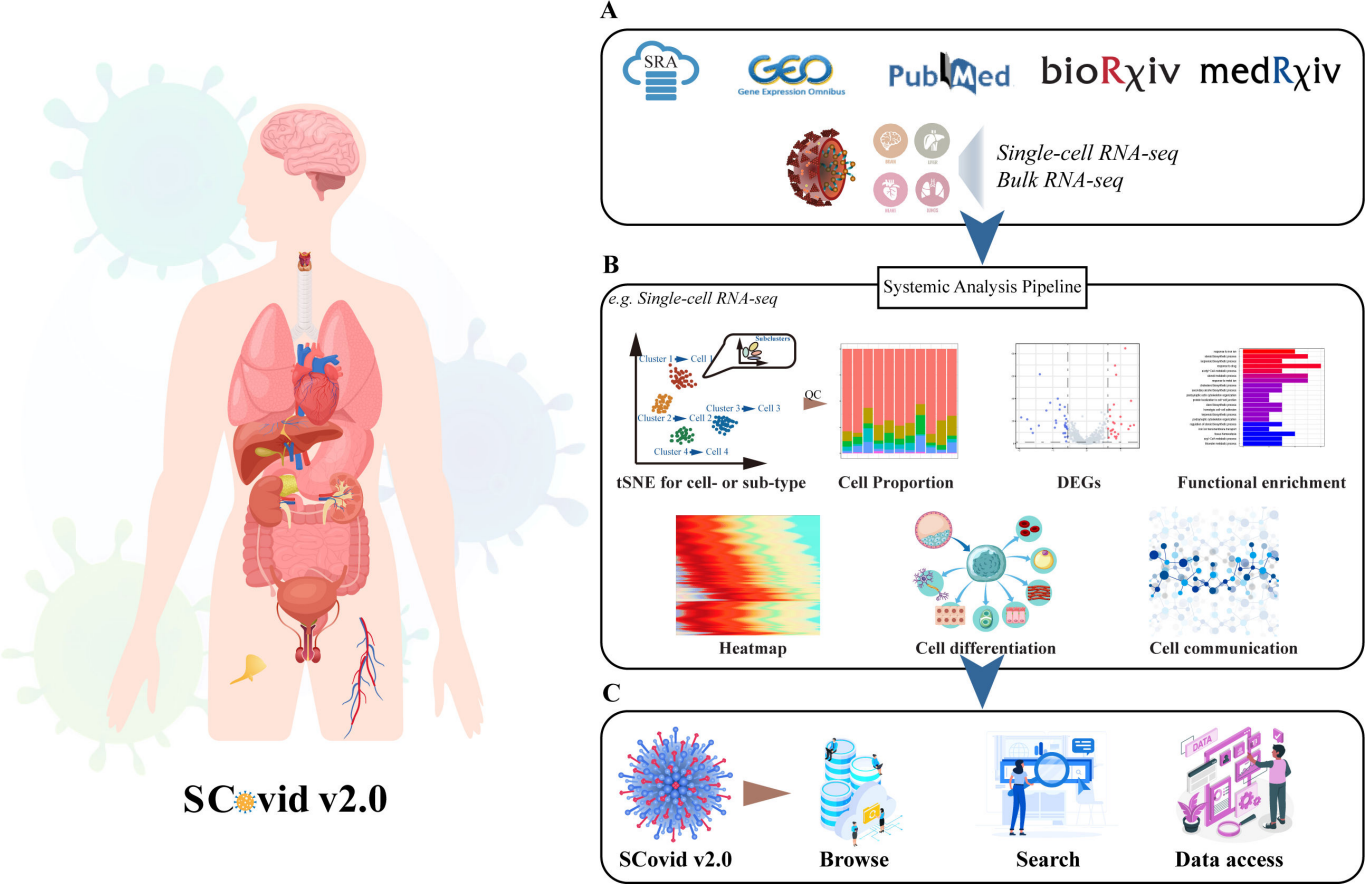
Overview of data collection, analysis workflow, and database function. (**A**) The source of SCovid data. (**B**) The workflow of SCovid. Here is an example of the single-cell RNA-seq workflow. (**C**) The functional modules of SCovid are a user-friendly web interface for users to browse data set information, search genes in single or multiple data sets, and acquire data sets.

The updated version of SCovid v2.0 contains more capabilities, like a body browser in the “Browse” interface and incorporates search engines in the “Search” interface to query the details regarding the relationships for DEGs. We offered a user-friendly web interface that allowed users to visualize the data sets in a few flexible steps. The schematic workflows of the SCovid v2.0 database are depicted in Fig. 2.

#### Browse data set

By clicking the hierarchical classification tree (e.g., Browse → Single-cell → Airway → Eddins et al. [endotracheal aspirates]), users can browse any project in the “Browse” page that we constructed. After selecting a data set, all the detailed and visual information, including “Detailed description,” “tSNE,” “Cell proportion,” “DEG description,” “Heatmap,” and “Cell differentiation” would be retrieved. In detail, the users can click the hierarchical classification tree of Eddins et al. (endotracheal aspirates). Additionally, the accession number and publication title contain hyperlinks that clients can follow, displaying detailed information on the data set.

##### Detailed description

Global information contains the paper title, accession, tissues, grouping, cell number, cell type, sample source, relevant publication information, and potential data corrections. The detailed description section consists of an overview of the data set, the tSNE dimensional reduction plot, cell components, heatmap of the hypervariable gene, cell differentiation trajectory, and cell- or sub-type interaction plot for cell- and sub-type.

##### tSNE

**Fig 3:**
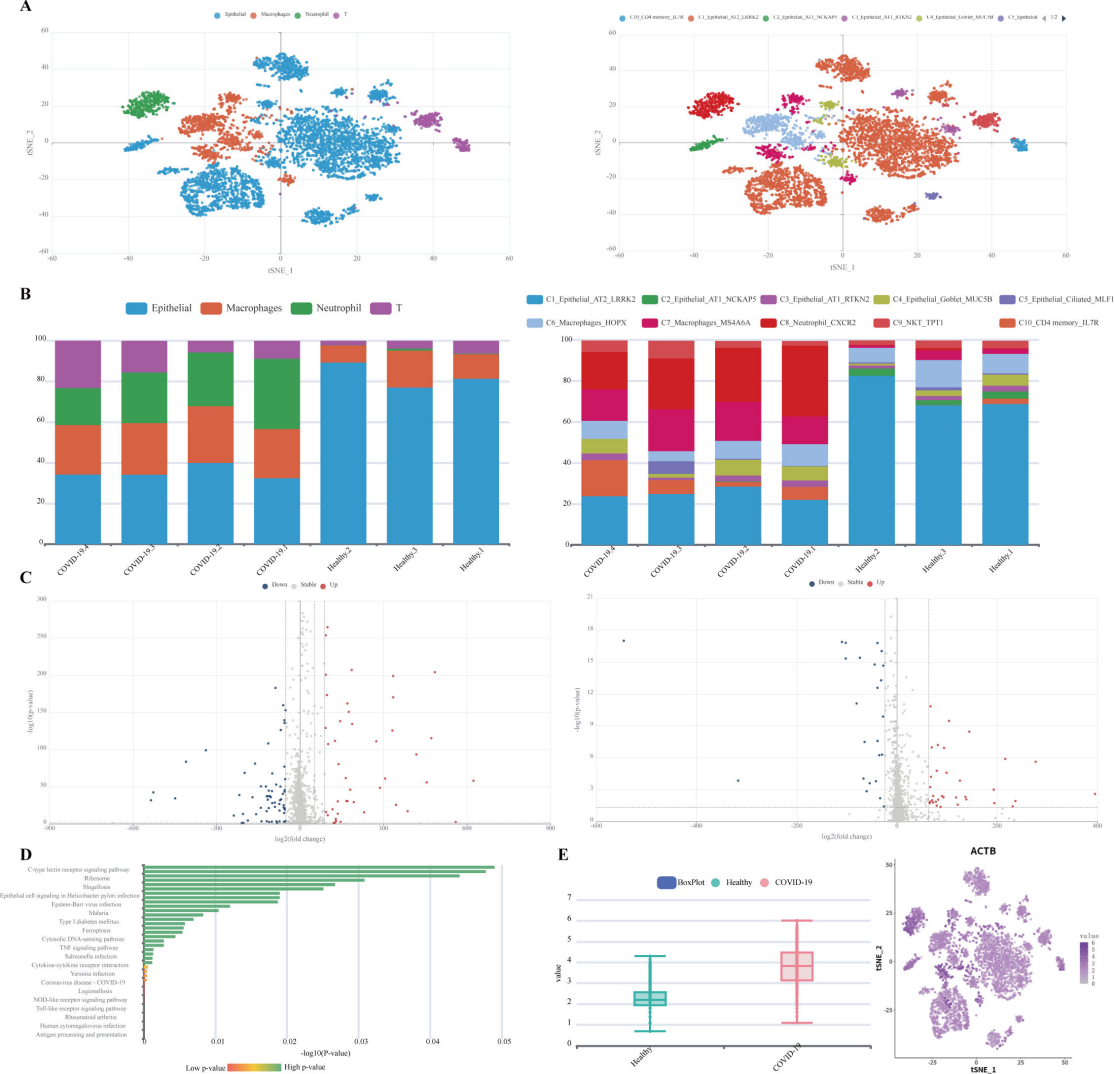
Database functions on the Browse page of SCovid v2.0. An example is the overview of GSE186267 (Eddins et al. [endotracheal aspirates]). (**A**) Two-dimensional tSNE plot. The colors of points represent the cell- (left) or sub-type (right) to which cells belong. (**B**) Cell proportion plot that displays the proportion of each cell- (left) or sub-type (right) per sample in the selected data set. (**C**) The volcano plot shows the statistically significant DEGs between COVID-19 and control. (**D**) KEGG classifications of downregulated genes (e.g., epithelial). The vertical axis shows the names of clusters of KEGG terms, and the horizontal axis displays the -Log_10_ (*P*-value). A *P*-value < 0.05 was used as a threshold to select significant KEGG terms. (**E**) Expression from each group and tSNE projection (e.g., ACTB).

Visualization of the selected data set using tSNE analysis is displayed in the “tSNE” section with colorful spots designating various cell- or sub-types (Fig. 3A).

##### Cell proportion

The “Cell proportion” section displays a bar plot to show the cell- or sub-type proportion across samples. Each bar represents a sample, and different colors stand for different cell types (Fig. 3B).

##### DEGs description

When clicking the cell type or sub-type in the “DEG description,” the list of significant DEGs, volcano plot, and GO/KEGG enrichment results will be displayed. The users can select the interested cell- or sub-type to browse the interactive information, including a volcano plot (Fig. 3C), a table, and GO/KEGG enrichment bar plots (Fig. 3D). When positioning the mouse on any bubbles of the volcano plot showing all stably expressed genes, the detailed information, including gene symbol, Log2FC, *P*-value, and change status, would pop up. The result table displays the statistically significant DEGs between COVID-19 and controls in the selected cell type of this data set. In the result table, clicking the “detail” link would lead to the detailed plots, including a violin plot and a tSNE projection plot for the specific gene (Fig. 3E). The GO enrichment and KEGG pathway bar plots display classifications of up-/downregulated genes, in which hovering over any bars would pop up detailed information, including term ID, term description, *P*-value, and gene symbol.

##### Heatmap

**Fig 4:**
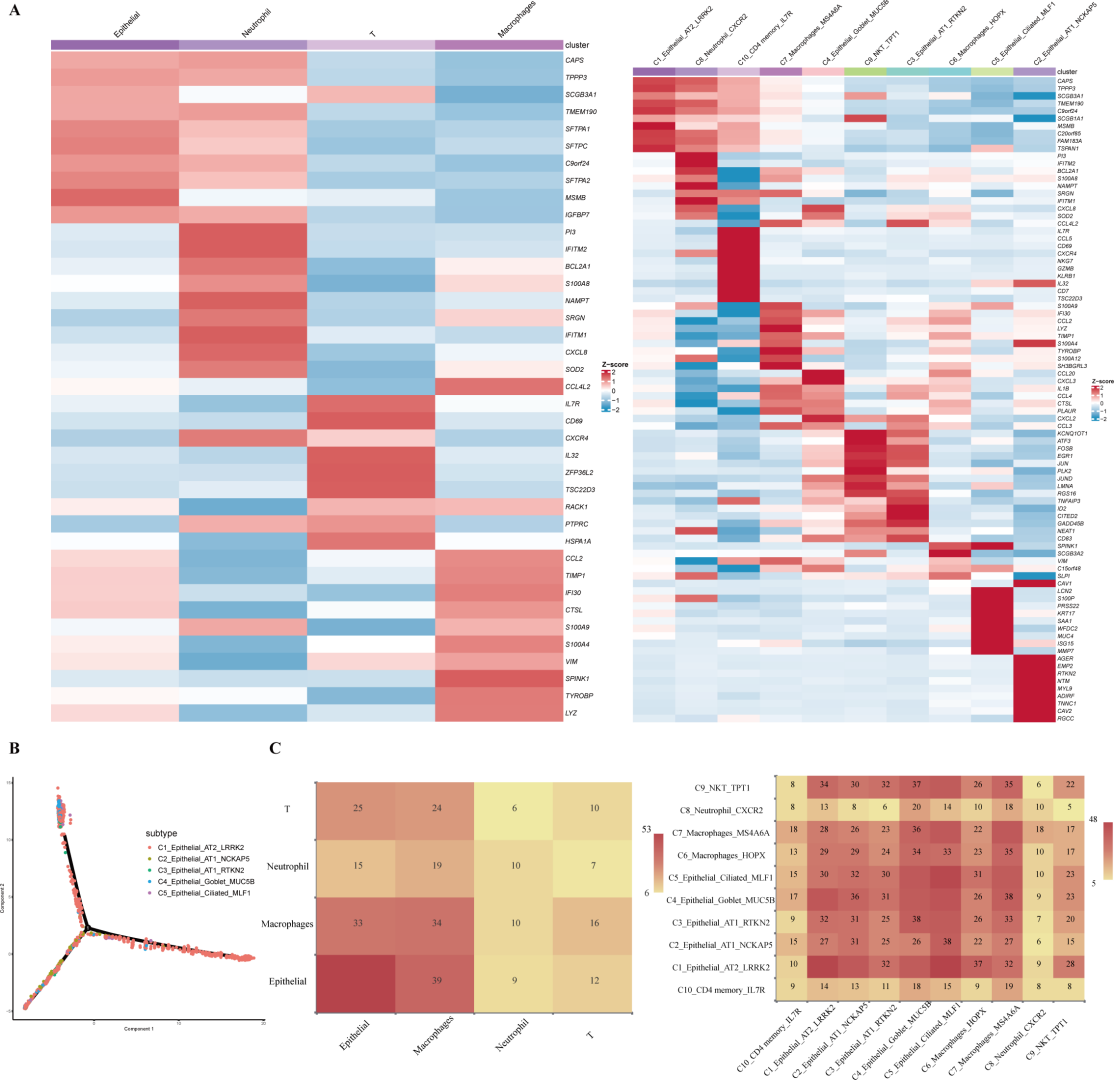
Downstream analysis on the Browse page of SCovid v2.0. (**A**) The heatmap shows the expression profile of high-variance genes in different cell- (left) or sub-types (right). (**B**) The cell differentiation plots show cell differentiation trajectories in cell-types (e.g., epithelial). (**C**) The two types of cell communication plots explain cell–cell interaction and interaction strength between different cell- (left) or sub-types (right).

The heatmap that displays the expression profile of high-variance genes in various cell- or sub-types is available in the “heatmap” section (Fig. 4A). The individual tiles in the heatmap are scaled with a range of colors proportionate to gene expression values. The gene sequences correspond to the matrix rows, and the cells correspond to the columns.

##### Cell differentiation

The cell differentiation section shows cell differentiation trajectories in different cell types (Fig. 4B).

##### Cell communication

The cell communication plots explain cell-to-cell interaction and interaction strength between different cell- or sub-types. We presented communication relationships in heatmaps and network maps (Fig. 4C).

### Search

On the “Search” page, SCovid v2.0 offers single- and multiple-gene searches. SCovid v2.0 allows users to enter its symbol to query its associated DEG information in tissue types, data types, and virus types. As mentioned above on the “Browse” page, a thorough table will be returned. In the current version of the search engine, many new filters have been added to meet the requirements of researchers from many disciplines, and users can restrict filters through multiple dimensions, thereby achieving more direct access to the associated data. In addition, we provided a functional module in the “Search” page to compare and visualize of gene expression levels between the data sets, tissues, and data types. This function can give users direction for specifically expressed genes across multiple data sets but cannot serve as a sole source to identify biological roles (17).

### Download

All data can be downloaded on the “Download” page, containing the gene expression profile, difference of all genes and markers (only for scRNA-seq data), and DEGs (only for bulk RNA-seq data).

### Submit

The “Submit” module is first established so that the user could contribute fresh data about COVID-19. Additionally, SCovid database will continue to be updated constantly to include more data.

### About

The “about” module provides detailed information about the database.

### Case study: the dysexpression pattern of IFNAR2 changes in different data, virus and tissue type

Recently, to investigate the mechanisms underpinning the dysfunctional immune response in SARS-CoV-2 infection, Edahiro et al. combined multi-omics data of >895,000 PBMCs from 73 COVID-19 patients and 75 healthy controls of Japanese ancestry with host genetic data (2). The biological and host genetic involvement of innate immune cells in COVID-19 severity was underlined by their investigation. In this work, the authors established that an IFNAR2-rs13050728 risk variation associated with COVID-19 showed context- and monocyte-specific expression quantitative trait loci effects. IFNAR2, a protein-coding gene involved in immune response or antiviral activity, has been linked to a novel form of life-threatening viral illness in coronavirus biology (18–20). Unfortunately, the differential patterns of expression for IFNAR2 in multi-data- and multi-tissue-types were undercharacterized.

To capture the highly heterogeneous expression process at high resolution, we searched IFNAR2 on the “Search DEGs in all conditions” of the “Search” page to completely ascertain the expression according to the default threshold (*P* < 0.05 and |log_2_FC| > 2). IFNAR2 was significantly expressed in two tissues (blood and heart) as well as four different cell types (T, Plasma, Macrophage, and NK) according to the findings (Table 2), thereby demonstrating tissue specificity by the discrepancy in the regulatory relationships between various tissues. Expression regulation pattern convergence existed in three single-cell RNA-seq data sets, including GSE171668, GSE178404, and GSE185457. We noticed significant variations in IFNAR2 among immune cells, especially lymphocyte sub-types, which may indicate a potential regulation pathway (Table 3). In addition, IFNAR2 differed across four tissues (intestine, lung, brain, and blood) in five bulk RNA-seq data sets (GSE149312, GSE152586, GSE164332, GSE171110, and GSE179850), revealing more subtle differences in the molecular mechanisms of COVID-19 (Table 4). Surprisingly, IFNAR2 varied across HCoV-229E and HCoV-OC43, suggesting the potential to guide a pan-coronavirus vaccine by targeting cross-reactive epitopes (Table 5) (21). When we searched IFNAR2 on the “Data comparison” of the “Search” page, we could visually see its gene expression levels in health or disease states within different data sets. While the results were highly correlated, visible variation among them highlights their complementary nature. These results demonstrated the utility of SCovid v2.0 in revealing the differential expression and distribution of genes in COVID-19.

**TABLE 2:** The cell-type differential expression of IFNAR2 in COVD-19 single-cell RNAseq data sets

Gene symbol	*P*-value	LogFC	Cell type	Tissue	Accession	Data set
IFNAR2	1.43e-117	−3.98	T	Heart	GSE171668	Delorey TM et al. (Heart)
IFNAR2	1.27e-09	2.07	Plasma	Blood	GSE178404	Lim et al. (PBMC)
IFNAR2	0	−3.11	Macrophage	Heart	GSE185457	Fukuma N et al. (Right ventricles)
IFNAR2	4.92e-195	−6.75	NK	Heart	GSE185457	Fukuma N et al. (Right ventricles)

**TABLE 3:** The sub-type differential expression of IFNAR2 in COVD-19 single-cell RNAseq data sets

Gene symbol	*P*-value	LogFC	Sub-type	Tissue	Accession	Data set
IFNAR2	6.61e-87	−4.28	C7_CD4_Tm_IL7R	Heart	GSE171668	Delorey TM et al. (Heart)
IFNAR2	0	−2.44	C6_Epithelial_AT1_LAMA3	Lung	GSE171668	Delorey TM et al. (Lung)
IFNAR2	2.8e-2	3.23	C7_Monocyte_FCGR3A	Blood	GSE178404	Lim et al. (PBMC)
IFNAR2	3.82e-09	2.08	C8_Plasma_IGHG3	Blood	GSE178404	Lim et al. (PBMC)
IFNAR2	1.06e-116	−4.54	C10_NK_KLRF1	Heart	GSE185457	Fukuma N et al. (Right ventricles)
IFNAR2	6.15e-24	−2.25	C11_NK_TCF4	Heart	GSE185457	Fukuma N et al. (Right ventricles)
IFNAR2	7.78e-24	−2.11	C12_NK_NEGR1	Heart	GSE185457	Fukuma N et al. (Right ventricles)
IFNAR2	3.86e-3	−10.14	C13_NK_KLRF1	Heart	GSE185457	Fukuma N et al. (Right ventricles)
IFNAR2	0	−3.12	C6_Macrophage_MRC1	Heart	GSE185457	Fukuma N et al. (Right ventricles)
IFNAR2	1.81e-93	−3.06	C7_Macrophage_SLC11A1	Heart	GSE185457	Fukuma N et al. (Right ventricles)

**TABLE 4:** The differential expression of IFNAR2 in COVID-19 bulk RNA-seq data sets

Gene symbol	*P*-value	LogFC	Tissue	Accession	Data set
IFNAR2	4.01e-4	−588.81	Intestine	GSE149312	Lamers MM et al. (Intestinal organoids)
IFNAR2	3.86e-2	4.28	Lung	GSE152586	Kobayashi Y et al. (AT2 cells)
IFNAR2	8.89e-3	−8.99	Brain	GSE164332	Gagliardi S et al. (Frontal cortex)
IFNAR2	1.62e-2	969.27	Blood	GSE171110	Lévy Y et al. (Whole blood)
IFNAR2	2.15e-4	8.41	Blood	GSE179850	Ebihara T et al. (Whole blood)

**TABLE 5:** The differential expression of IFNAR2 in other human coronaviruses bulk RNA-seq data sets

Gene symbol	*P* value	LogFC	Virus type	Accession	Data set
IFNAR2	5.62e-3	−4.37	HCoV-229E	GSE159513	Trimarco J et al. (Cells)
IFNAR2	8.86e-07	−12.32	HCoV-229E	GSE197644	Zhu X et al. (Cells)
IFNAR2	9.68e-06	19.91	HCoV-OC43	GSE198398	Feng Y et al. (Cells)

## DISCUSSION

The SCovid database’s initial release, SCovid v1.0, had only limited single-cell RNA-seq data collected across 10 human tissues. The number of transcript data related to COVID-19 has grown significantly in recent years as a result of the advancement of high-throughput sequencing technology. The importance and high demand of molecular processes for COVID-19 indicate the urgent need to compile the corresponding data sets and update SCovid. The data set and functionality of SCovid v2.0 are considerably enhanced. For instance, we reconstructed the single-cell analysis pipeline in v2.0, which includes cluster analysis, differential expression analysis, cell communication, and cell differentiation of cell- and sub-types. Further investigating the molecular processes of COVID-19, we also constructed a bulk RNA-seq pipeline and supplied interactive charts for viewing.

The main challenge in SCovid 2.0 is to systematically compare molecules between the data sets, tissues, data types, and even virus types. Here, we adopted a standardized pipeline to integrate data sets, including bulk RNA-seq for coronaviruses and scRNA-seq data for COVID-19. For example, we uniformly converted bulk RNA-seq count data into TPM data to avoid the problem of sequencing depth or sample size, thus facilitating researchers to assess differences in gene expression levels under different conditions more accurately. From a global perspective, users can search the genes they are interested in and further compare their molecular characteristics in all conditions; and from a local perspective, SCovid v2.0 allows users to conduct a comparative analysis of gene expression levels across data sets within different tissue types. This module enables the decoding of the functional implications associated with differential molecules. In SCovid v2.0, the biological function of several molecules has been validated in previous studies, such as IFNAR2, as a protein-coding gene involved in immune response or antiviral activity.

Another obvious challenge is the non-standard cell typing of scRNA-seq data. First, it can be challenging to compare and integrate data from different studies since the same cell type may be referred to by different names across studies, which leads to ambiguity and confusion in downstream analysis. Second, non-standard cell naming results in inaccurate cell type classification and affects the interpretation of the results. Third, it can make it challenging to identify rare cell populations and subtypes that may be critical for understanding the underlying biology of the system being studied. Finally, non-standard cell naming can hinder the reproducibility of the study, making it difficult for other researchers to validate the findings. Therefore, based on the cell annotation method in the original paper, we have systematically applied a consistent nomenclature in scRNA-seq data to ensure data quality, comparability, and reproducibility. Indeed, this work has proven to be highly effective in uncovering the molecular characteristics and data insights of COVID-19 and has significantly facilitated further research in this area.

SCovid v1.0 has provided an unprecedented benchmark set of COVID-19, while v2.0 further expands and refines the benchmark set. In the future, our team will devote maximum efforts to establishing a thorough framework to investigate the pathogenic mechanism of COVID-19, thereby directing clinical transformation. Indeed, with the emergence of abundant publications and various sequencing data, we will continue to maintain and update the SCovid database with additional data sets and web tools. We will update and integrate more data sets into SCovid v2.0: (i) continuously mining data sets to expand the current molecule atlas. Compared with SCovid v1.0, we have added a “Submit” module that allows users to submit relevant data sets. According to the data submitted by the “Submit” module and GEO database, we will update and maintain the database every 6 months. (ii) Expanding the tissue and virus type based on existing data. For example, the influenza virus will be added in future versions of SCovid to provide users with more comprehensive information. (iii) integrating additional analytical modules to provide a richer understanding of the molecular mechanism. We will also update a few integrated RNA-Seq web tools. Based on prior literature and experimental validation, for instance, we will incorporate the COVID-19 protein interaction module. (iv) summarizing data across studies, per tissue, or whole. On the basis of data boom, we will add reasonable and effective integration strategies. In summary, we anticipate that the SCovid v2.0 database will be an essential resource for research on human coronaviruses in the future.

## Data Availability

The data that support the findings of this study are openly available in SCovid v2.0, which is freely accessible at http://bio-annotation.cn/scovid or http://bio-computing.hrbmu.edu.cn/scovid/. The R codes used to generate data sets in SCovid were shared on Github (https://github.com/GuoYouHe/ScovidV2.0). All software tools used in this study are freely available.
